# Genome-wide screening of microsatellites in golden snub-nosed monkey (*Rhinopithecus roxellana*), for the development of a standardized genetic marker system

**DOI:** 10.1038/s41598-020-67451-2

**Published:** 2020-06-30

**Authors:** YanSen Cai, HaoYang Yu, Hua Liu, Cong Jiang, Ling Sun, LiLi Niu, XuanZhen Liu, DaYong Li, Jing Li

**Affiliations:** 1 Key Laboratory of Bio-Resources and Eco-Environment (Ministry of Education), Sichuan Key Laboratory of Conservation Biology on Endangered Wildlife, College of Life Sciences, Sichuan University, Chengdu, 610064 People’s Republic of China; 2 Department of Cell Biology and Genetic, School of Basic Medical Sciences, Southwest Medical University, Luzhou, 646000 People’s Republic of China; 3 Chengdu Zoo, Chengdu, 610064 People’s Republic of China; 4 College of Life Sciences, China West Normal University, Nanchong, 637000 People’s Republic of China

**Keywords:** Genetics, Molecular biology, Zoology

## Abstract

Golden snub-nosed monkey (*Rhinopithecus roxellana*) is an endangered primate endemic to China. The lack of standardized genetic markers limits its conservation works. In the present study, a total of 1,400,552 perfect STRs was identified in the reference genome of *R. roxellana*. By comparing it with the 12 resequencing genomes of four geographical populations, a total of 1,927 loci were identified as perfect tetranucleotides and shared among populations. We randomly selected 74 loci to design primer pairs. By using a total of 64 samples from the Chengdu Zoo captive population and the Pingwu wild population, a set of 14 novel STR loci were identified with good polymorphism, strong stability, high repeatability, low genotyping error rate that were suitable for non-invasive samples. These were used to establish a standardized marker system for golden snub-nosed monkeys. The genetic diversity analysis showed the average H_O_, H_E,_ and PIC was 0.477, 0.549, and 0.485, respectively, in the Chengdu Zoo population; and 0.516, 0.473, and 0.406, respectively, in Pingwu wild population. Moreover, an individual identification method was established, which could effectively distinguish individuals with seven markers. The paternity tests were conducted on seven offspring with known mothers from two populations, and their fathers were determined with high confidence. A genotyping database for the captive population in the Chengdu Zoo (n = 25) and wild population in Pingwu country (n = 8) was acquired by using this marker system.

## Introduction

Golden snub-nosed monkey (*Rhinopithecus roxellana*) is an endangered Old World Monkey that is endemic to China, and it is currently under the National Protection level I^1,2^. Alongside the giant panda, the golden snub-nosed monkey is known as ‘China’s national treasure’ and is often cited as one icon of the national biodiversity conservation. These monkeys are threatened by large-scale forest shrinkage and ecological environment deterioration at present. The wild population is estimated at ~ 15–22 thousand in total, and now only occur in three isolate regions of temperate alpine forests: Sichuan-Gansu mountains (~ 66.7%), Qinling mountain (~ 26.6%), and Shennongjia mountain (~ 6.7%)^2^. Meanwhile, more than 452 captive individuals were kept in 44 institutions nationwide by the end of 2018^3^. Although the international community and the Chinese government have made great efforts to protect this precious species, some urgent issues remain unresolved.

At present, the conservation strategies of golden snub-nosed monkeys mainly include the protection of the wild population and the protection of the captive population. They are both limited by the lack of a standardized genetic marker. Microsatellites, also known as short tandem repeats (STRs) and simple sequence repeats (SSRs), are a well-known tool for genetic diversity analysis. Although STR loci analysis has been used to assess the genetic variability and population size for golden snub-nosed monkeys by some researchers, there are still some unresolved problems.

One problem is that different loci were applied to different populations, which makes it difficult to evaluate genetic diversity among populations. Most of the golden snub-nosed monkey loci in previous studies were screened from the loci of related primates, such as rhesus monkeys and human^4–8^. For example, Pan (2005)^4^ analyzed the genetic diversity of three golden snub-nosed monkey populations using 14 microsatellite loci. Later, Chang (2012a)^5^ and Zhou (2018)^6^ used 16 loci and 12 loci to study the Shennongjia population, respectively. Most of these loci were different from those in Pan’s study, which made it difficult to compare and analyze among populations based on their findings and limited the formulation and implementation of conservation strategies. Furthermore, the lack of standardized genetic markers also makes it impossible to compare the genetic diversity within the same population of different periods. In this case, the two studies on the Shennongjia population seem to indicate that the genetic diversity of this population was the lowest among all golden snub-nosed monkey populations, but it was hard to say whether the diversity had increased or decreased after decades of conservation work.

Secondly, most of the loci in previously studies were not developed for non-invasive sampling, which can easily subject to mistyping during PCR amplification, especially in fecal samples or degraded tissue samples with low quality or concentration of template DNA. Unfortunately, the non-invasive fecal samples are much easier to obtain than blood and tissue samples, and it is a more practical sampling method in the wild. Therefore, there is still a lack of STR markers that can be widely applied to non-invasive samples.

Moreover, previous studies did not consider the needs of captive populations. In captive breeding, due to the factors such as unclear genetic background, very small founder population, irregular and incomplete genetic lineage records, inbreeding was very likely^9^. In addition, breeding programs in the zoo and between zoos can only rely on their incomplete pedigree records at present, lacking standardized genetic markers to support and validate these records. Breeding plans based on defective pedigree records are likely to lead to a series of problems, such as inbreeding, subspecies hybridization, loss of genetic diversity and population degradation^3^. In addition, the previous loci have not been tested for validity in captive populations with a very small population size. Therefore, it is necessary to develop a standardized marker system and to establish an accurate paternity test method and a clear genetic pedigree record for captive populations.

The traditional ways of STR development involve time-consuming, costly and labor-intensive approaches, such as magnetic beads enrichment and Random amplification of polymorphic DNA (RAPD). Fortunately, the availability of golden snub-nosed monkey high throughput sequencing genomes provide us with the opportunity for genome-wide mining STRs and discovery of polymorphic loci across geographic populations. This method is much cheaper, more effective and more successful than traditional methods. In this study, by genome-wide screening for STRs in the golden snub-nosed monkey and comparing it with 12 available resequencing genomes, we are committed to the development of tetranucleotide STR loci with high cross-population polymorphism, strong stability, good repeatability, low genotyping error rate and, most importantly, suitability for non-invasive samples. This standardized marker system in golden snub-nosed monkeys could be widely used in individual identification, pedigree validation, genetic diversity analysis and development of conservation strategies for both wild and small, captive populations.

## Results

### Development of STR markers

**Figure 1:**
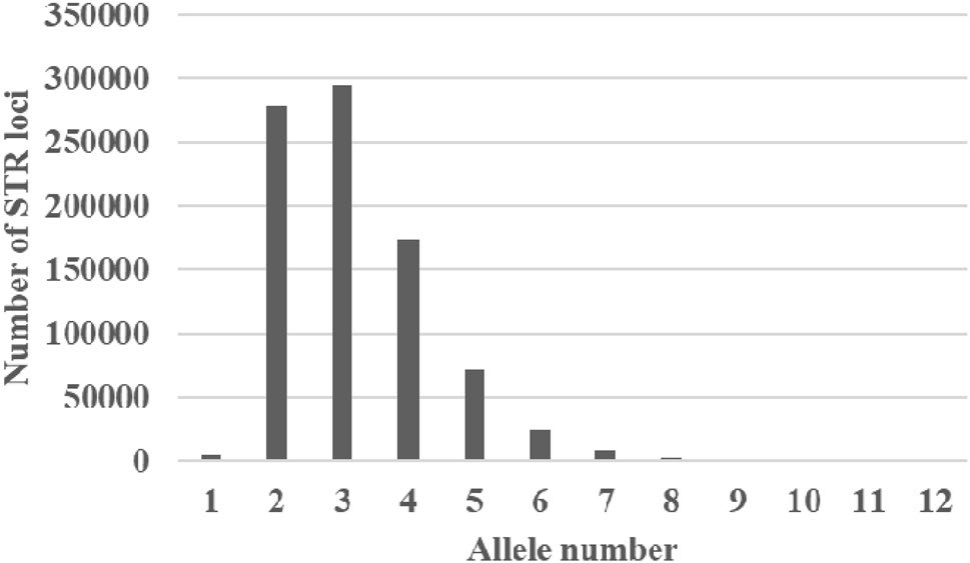
Distribution of STR alleles based on 12 sequencing data.

A total of 1,400,552 perfect STRs (ranging from mononucleotide to hexanucleotide) was identified in the golden snub-nosed monkey genomic data. By comparing them with the 12 resequencing genomes, a total of 864,838 shared STRs were obtained (Fig. 1). The STRs with three alleles were the most abundant polymorphic STRs, with the number of 295,265. The total number of STRs with alleles greater than or equal to 3 was 580,900, from which 2,435 loci were tetranucleotide.

Further analysis found that 508 of the 2,435 loci extracted in the reference genome contained defective alleles in the 12 resequencing genomes. Therefore, these loci were excluded in subsequent analysis. Finally, based on our selection criteria, a total of 1,927 candidate polymorphic loci were left, with the repeat number range from 1 to 18. Seventy-four of them were randomly selected to design primers by Primer Premier 6.0. As a result, 69 loci were successfully designed.

We randomly synthesized 42 of the 69 primer pairs and used them to amplify target STRs in the DNA of both blood/tissue and fecal samples. After amplification, 31 primer pairs showed a single band of the expected product and were labeled with fluorescent signals on their forward primer 5′ terminals (FAM or HEX) and tested for polymorphism. Finally, 16 primer pairs were chosen that showed both with a good polymorphism in their loci and with excellent amplify ability on both fecal and blood/tissue DNA (details in the “Methods” section). Our STR sequences were unique when compared to those for the golden snub-nosed monkey in NCBI. The sequence data of the 16 loci were submitted to NCBI (GenBank accession number: MN094891–MN094906). The 16 novel tetranucleotide STRs discovered for golden snub-nosed monkeys were shown in Table 1.

**Table 1:** Characteristics of the novel STR marker system.

Locus	Motif	Primer pair (5′-3′)	Size (bp)	Location
GSM03	CAAA	F: TTCTCCTTCTCTGACACATC R: TACTGCCAAGTTAGAGTGAG	154–166	Non-coding region
GSM04	CAAA	F: TTGCAGTGAGCCAGGATAGC R: TTACCTATGAGTGCCAGGCC	171–187	Intron
GSM05	CTTT	F: TAACTCACTGGAGCAGAGA R: GCAGTAAGCCAAGATCGT	145–149	Intron
GSM07	TAAA	F: ATGATCACACCACTGCACCC R: TTCTTCAAGGCTGTGTCCCC	140–152	Non-coding region
GSM13	ATAC	F: CTGGGCGATAGAATGAGACCC R: GGTCCATGGCTCTTAAGGGG	125–137	Intron
GSM16	TAGA	F: TTATCGATGGCTCAGCTGGC R: ACAAATGGGTGTTTGGGTGG	190–194	Non-coding region
GSM18	CAAA	F: AGCGGGAGAATCACTTGAGC R: TGTTTTTGGTTTGGGGGTTTGG	160–188	Intron
GSM21	CAAA	F: GCAGGTGGAATGCTTGAACC R: GCATCTTGCATCTGCTGAGC	131–155	Non-coding region
GSM25	GAAT	F: TGCTTCTAGTGTTTTGCAGTGC R: GCTGGGCATTAGTGGAGAGG	129–137	Non-coding region
GSM31	TTTA	F: ATGGTTGAGCAAGCCCAGG R: TGATCCCAGAGGTTTGAGGC	157–165	Intron
GSM32	ATTT	F: GGTCTGCTTGTTGAATATGGAGC R: CACACCACTACACTCCAGCC	168–188	Intron
GSM42	ATGA	F: GAAACCAAACTGCCCACACC R: CACAAAACACCACAGACCGG	108–128	Intron
GSM47	TAGA	F: TCTAGCCTCCAGAACCAT R: CCTATGTATCTATCTGCCTATC	155–175	Intron
GSM51	CAAA	F: ATCTGGACTGCTTATTCTGT R: AGGAAGGCTTCATACTCAAG	120–128	Non-coding region
GSM69	TCTT	F: GAGCTAAGTTGTATATTCTGGCTCC R: CACACCACTGTACTCCAGCC	134–154	Non-coding region
GSM75	TACA	F: AGATGAGATGGTGCCAATG R: GCCATGCAGGTTGTAGATA	114–130	Non-coding region
ZFX-ZFY		F: TTATGGTGAAAGCCAAGAA R: GCAATTTCAGCAACATCTAAG	X-211 Y-140	

### Sex identification

**Figure 2:**
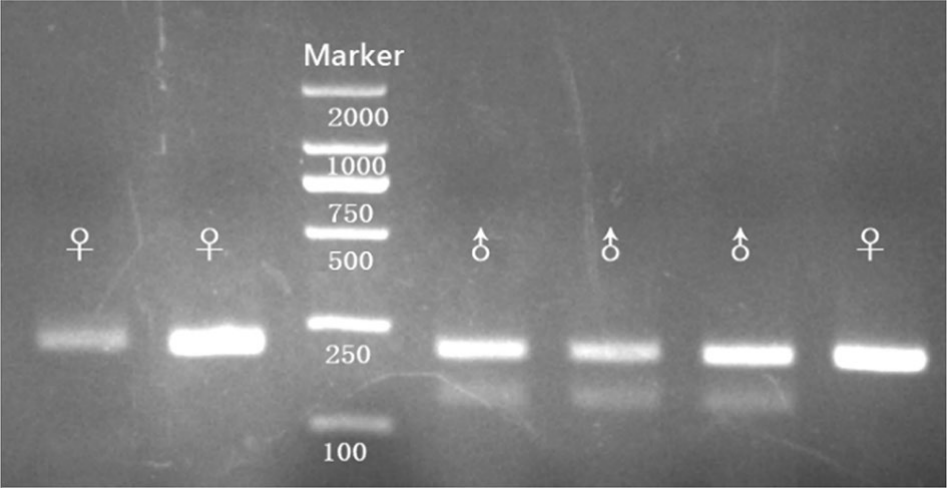
Gel images showing the results of the sex identification for golden snub-nosed monkey in fecal samples. Males were all identified by two bands (211 bp and 140 bp) and the females were all identified by one single band (211 bp).

We designed a sex identification primer pair ZFX-ZFY. Males can be identified by two bands (211 bp and 140 bp) while females can be identified by one single band (211 bp) (Fig. 2). All 64 of our samples were tested, and a total of 28 males and 36 females were easily and correctly identified. For duplicate samples (one individual sampled multiple times) in genotyping, their genders were checked and confirmed to be the same. The sex ratio M/F for the captive and wild population was 1.08 (13:12) and 0.69 (9:13), respectively.

### A standardized genetic marker system based on polymorphic STRs

The goal of a standardized genetic marker system for golden snub-nosed monkey is to be applicable for both captive and wild populations, therefore the chosen loci must be suitable for noninvasive samples. Alongside the 22 fecal samples from the Chengdu Zoo, 30 long-term exposed fecal samples from the wild were used to test the sensitivity and quality of the marker system. As a result, all 16 markers showed 100% success rates on amplifying the 52 fecal samples and the repeat tests showed no multiple amplification or false amplification exist, which indicated that they respond very well to fecal samples and could be reliably used for fecal DNA analysis. Moreover, by comparing the genotypes of the 16 loci between fecal DNA and blood DNA of the same individual in the captive population, no difference was found between them.

In the wild samples, the tests on the relationship between the exposure time of fecal samples and the stability of the 16 loci showed that the marker system could be used for fecal samples exposed to field environment for up to one week (Supplementary Table 2). Subsequently, the 16 markers were used to genotype all our samples and identify individuals to build a data base for golden snub-nosed monkeys in Chengdu zoo and Pingwu (Supplementary Table 3).

**Table 2:** The genetic diversity of golden snub-nosed monkeys in the Chengdu Zoo captive population and Pingwu wild population.

Locus	Chengdu Zoo						Pingwu wild						Total H_E_^a^
	k	N	HWE	H_O_	H_E_	PIC	k	N	HWE	H_O_	H_E_	PIC	
GSM47	6	25	0.007	0.647	0.781	0.738	3	22	0.016	0.818	0.627	0.557	0.735
GSM42	6	25	0.004	0.618	0.774	0.728	5	22	0.082	0.500	0.481	0.436	0.684
GSM32	3	25	0.517	0.588	0.581	0.481	4	22	0.538	0.591	0.464	0.403	0.673
GSM04	5	25	0.469	0.588	0.659	0.585	3	22	0.243	0.818	0.635	0.549	0.667
GSM21	5	25	0.130	0.706	0.73	0.672	3	22	0.541	0.318	0.321	0.292	0.618
GSM25	4	25	0.001	0.353	0.451	0.388	3	22	0.689	0.727	0.667	0.579	0.588
GSM51	3	25	0.722	0.529	0.593	0.496	2	22	0.075	0.773	0.585	0.500	0.588
GSM75	5	25	0.235	0.647	0.698	0.625	3	22	0.094	0.136	0.212	0.197	0.587
GSM31	3	25	0.511	0.676	0.578	0.48	3	22	0.873	0.500	0.517	0.451	0.569
GSM13	4	25	0.089	0.529	0.553	0.469	4	22	1	0.273	0.253	0.236	0.561
GSM69	3	25	0.042	0.324	0.412	0.370	3	22	1	0.455	0.495	0.367	0.518
GSM18	5	25	0.217	0.559	0.576	0.505	3	22	0.620	0.364	0.444	0.340	0.494
GSM05	2	25	0.013	0.294	0.500	0.372	2	22	0.272	0.500	0.384	0.305	0.472
GSM03	4	25	0.605	0.206	0.240	0.220	3	22	0.085	0.455	0.571	0.490	0.450
GSM16	4	25	0.043	0.088	0.142	0.136	2	22	0.405	0.500	0.477	0.428	0.319
GSM07	2	25	1	0.176	0.163	0.148	1	22					0.121

*k* number of alleles, *N* number of individuals, *HWE* Hardy–Weinberg equilibrium P value, *H*_*O*_ observed heterozygosity, *H*_*E*_ expected heterozygosity, *PIC* polymorphic information content

^a^Total H_E_ was calculated from all of the 47 individuals.

**Figure 3:**
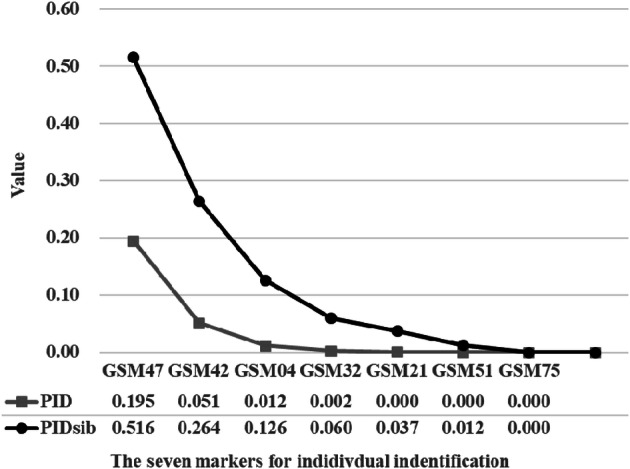
Cumulative PID and PIDsib values for the different number of STR combinations. Loci were arranged according to their H_E_ value from high to low. The loci with the highest H_E_ value were selected and added one by one, and their PID and PIDsib values were calculated.

**Table 3:** Results of paternity analysis for 7 golden snub-nosed monkey offspring.

Offspring ID	Father ID	Number of analyzed loci	Pair loci mismatching	Trio loci mismatching	Trio LOD
G1	CC	14	0	0	12.01
	Other 8 captive population candidates	14	2 to 7	2 to 9	2.83 to − 28.05
G6	C16	14	0	0	10.98
	Other 8 captive population candidates	14	0 to 7	2 to 8	− 11.01 to − 29.89
J5	C16	14	1	2	2.94
	Other 8 captive population candidates	14	1 to 7	3 to 7	− 4.07 to − 23.79
Z1	A180	14	2	2	− 3.41
	Other 8 captive population candidates	14	1 to 5	3 to 6	− 4.71 to − 18.89
Y1	CC	14	0	0	4.74
	Other 8 captive population candidates	14	0 to 6	1 to 7	3.30 to − 24.30
H3	A180	14	0	0	12.83
	Other 8 captive population candidates	14	2 to 7	4 to 9	− 8.74 to − 31.65
PW15	PWA	14	0	0	2.54
	Other 8 wild population candidates	14	1 to 3	1 to 3	2.39 to − 9.06

### Genetic diversity

In the Chengdu Zoo captive population, the total number of alleles was 64, and the number of alleles per locus ranged from 2 to 6 with an average of 4 (Table 2). The observed and expected heterozygosity ranged from 0.08 to 0.72 and 0.154 to 0.803, with an average of 0.445 and 0.535, respectively. The PIC (Polymorphic information content) ranged from 0.147 to 0.758 with an average of 0.466. The results of the null allele test showed that the average F(Null) of the 16 loci was 0.0831, range from -0.0532 (GSM51) to 0.2633 (GSM13), and no signs of null allele. The HWE test showed six loci (locus 5, 16, 25, 42, 47 and 69) deviated from Hardy–Weinberg equilibrium (P < 0.05). We also tested the linkage disequilibrium of these loci, and the results showed that locus GSM31 and GSM32 were likely linked. Meanwhile, no homologous sequences of other loci were detected.

In the Pingwu wild population, due to a lack of polymorphism, Locus GSM07 was not included, so only 15 loci were involved in the analysis (Table 2). As a result, a total of 64 alleles were identified, and the number of alleles per locus ranged from 2 to 5 with an average of 3.13. The observed and expected heterozygosity ranged from 0.136 to 0.818 and 0.212 to 0.667, with an average of 0.515 and 0.476, respectively. The PIC ranged from 0.197 to 0.579 with an average of 0.409. None of the loci showed a null allele. One locus (GSM47) deviated from HWE (P < 0.05) in this population.

For GSM32 and GSM31, we suggest to keep GSM32 only, because it have one additional allele in the wild population and the higher total H_E_. We also recommend that GSM07 could be excluded from future genetic diversity analysis work for its low polymorphism.

In the end, only 14 loci were reserved in the standardized marker system. These 14 loci were used to analyze and compare the genetic diversity of captive and wild populations. The H_O_, H_E_ and PIC in captive population were 0.477, 0.549 and 0.485 respectively, and in the wild population were 0.516, 0.473 and 0.406 respectively. The average PIC in the captive and wild populations (0.485 vs. 0.406) showed that the genetic diversity of the captive population was slightly higher than that of the wild population.

### The individual identification

The PID and PIDsib value was calculated by Cervus 3.0.7^10^ to estimate the number of markers needed for individual identification (Fig. 3). The results showed that a minimum of 5 loci was required to reach PID < 0.001, while a minimum of 7 loci was required to reach PIDsib < 0.01. The seven STR markers were GSM04, GSM21, GSM32, GSM42, GSM47, GSM51 and GSM75. Further, the individual identification system was conducted on all of our samples. The result showed that the 64 samples belonged to 47 individuals which were the same as the identification results based on all 14 markers. These results revealed that our markers could distinguish members with highly similar genetic backgrounds, and could provide an effective identification method for both captive and wild golden snub-nosed monkeys.

### Paternity test

Based on the 14 novel STR markers, the biology parent–offspring relationships were compared with the records. In the captive population, all nine adult males were selected as candidate fathers for six offspring with known mothers. A total of 54 parent pairs were compared (Supplementary Table 4a). To determine the confidence in the assignment of parentage to the most likely candidate parent, the Trio LOD score was calculated by Cervus 3.0.7 (Table 3). The results showed that the recorded fathers were all supported to be the genetic fathers. Although two recorded fathers mismatched one (GSM05) and two loci (GSM05 and GSM47) with their offspring respectively, their Trio LOD scores and matched loci were the highest among all candidates (Table 3, Supplementary Tables 4a and 5).

In the wild population, a total of nine males in the wild population were selected as the candidate father. As a result, the dominant male was identified as the father of the cub to be tested, which was supported with high confidence (Trio LOD = 2.54), and all the 14 loci were matched. Meanwhile, no other candidate father was supported (Trio LOD range from 2.39 to − 9.06), their mismatch number of analyzed loci range from 1 to 3 (Table 3, Supplementary Table 4b).

We also tried a few other loci combination for paternity testing, using all 16, 13 (without GSM07, 31 and 05), 12 (without GSM07, 31, 05 and 47) and 7 (only individual identification loci were involved) loci respectively. The results showed that the 14 loci combination seemed to be the best, its results were very similar to those of 16 loci combinations (Supplementary Table 4a and 4b). The results of the 13 loci combination were also good, and all recorded fathers got positive Trio LOD. However, due to the fact that all the loci of two fathers of Y1 were completely matched, a candidate father (B27) achieved a higher level of Trio LOD and Trio Confidence than the recorded father (Supplementary Table 4c). The results of 12 and 7 loci combination were similar to that of the 13 loci combination, and two similar cases were found respectively (Supplementary Table 4d and 4e). According to the management records of the Chengdu Zoo, none of the candidates who got higher scores could actually be the true father of those offspring. Therefore, we believed that the combination of 13, 12 and 7 was not as good as the combination of 14 loci.

## Discussion

### The development of novel STR marker system

#### The standardized STR marker

Golden snub-nosed monkey is one of the world’s threatened primates and urgently needs protection. In recent years, researchers have used more and more STR markers for population genetic studies^4–8,11^, but they have been unable to reach consensus on the selection and application of the markers throughout. In this study, by genome-wide screening STR in golden snub-nosed monkey and comparing it with 12 resequencing genomes, we developed a standardized STR marker system based on 14 novel loci. These loci were found to have good polymorphism and amplified well, even on fecal DNA from both wild and captive populations.

We initially developed 16 loci however, two of them were rejected. Locus GSM07 had a very low polymorphism in the analysis of genetic diversity, indicating that it was not a very effective genetic marker. Loci GSM31 and 32 were linked, therefore, only one of them should be retained in a study. Here, we suggest that GSM07 and 31 should be rejected and the remaining 14 loci retained for the standardized marker system.

#### Applicability of non-invasive sampling

One great challenge in the genetic research for endangered species is that it is challenging to get samples with high-quality DNA. Basically, tissue samples can only be obtained during post-mortem autopsy. Blood sampling is relatively easy in captive breeding, but involves a complex collection process and may cause health hazards, which result in issues of animal welfare and ethics, and is therefore used with caution. As for wild monkeys, it is impossible to collect enough blood/tissue samples needed for genetic research. Non-invasive samples, such as shed hair and feces, are a valid alternative. However, the DNA quality or concentration of non-invasive samples is usually not very good, which may increase the rate of genotyping errors such as allelic dropouts or false alleles in genetic research. The non-negligible error rate in many laboratories is usually range from 0.2% to 15% per locus^12^, while higher error rates are known to occur in studies involving non-invasive samples with poor DNA quality or low concentrations (less than 0.5U), reaching an astonishing rate 50%^13^. Therefore, the loci selected to establish the standard STR marker system should be stable enough to produce minimal genotyping error and sensitive enough to respond to non-invasive samples.

In previous studies of golden snub-nosed monkey, most of the STR markers were screened from the STRs of other primates. These markers lacked response tests on non-invasive samples, which resulted in large number of markers being discarded due to failure in fecal DNA amplification (apart from lack of polymorphism) after population replacement. In this study, the sensitivity tests of our novel markers had a 100% amplification success rate in all 52 non-invasive DNA samples. Repeatability tests conducted by comparing the genotypes of fecal DNA and blood/tissue DNA showed no difference. Although the sample size of these tests was relatively small, the results showed that the selected markers had good potential in stability and reliability. Moreover, the tests on the relationship between the exposure time of fecal samples and the stability of these novel markers indicated that these markers could be used in wild fecal samples with an exposure time of one week. From here we see that this novel STR marker system is suitable for non-invasive sampling.

### The application of novel STR marker system

#### Individual identification

Based on the 14 novel markers, we established the genotype database of Chengdu Zoo golden snub-nosed monkeys. This database can be used to effectively identify individuals of golden snub-nosed monkeys, evaluate genetic lineage records and guide breeding. It was observed that the seven loci combination was highly suited for individual-level identification, with a PID value suggesting that 1 in 30,000 unrelated individuals will share the same genotype. Considering that the total number of individuals in this endangered monkey was far less than 30,000^2^, our markers were indeed enough.

#### Paternity test

In the paternity test, the results showed that the 14 loci combination was adequate for parentage analysis of both captured and wild golden snub-nosed monkeys. Although two record fathers showed mismatched loci with their offspring, such mismatches may be related to PCR errors or germline mutations. Many researchers believe 6–12 STR markers are enough for individual identification and parentage assignment, and too many markers may increase genotyping errors and overestimations of population sizes^14–16^. Even if the rate of typing error is low, mismatches are relatively common. According to Kalinowski et al.^10^, in a paternity test with 10 loci, if the rate of typing error is 1% there is a 26% chance that one or more of those single-locus genotypes is mistyped. In our study, 14 STRs were applied, which may potentially increased the genotyping error. In addition, in the two cases of mismatch, offspring only had fecal samples. The lower DNA quality and template concentration in non-invasive samples also could lead to an increase in PCR error rate. On the other hand, it is known that the length and polymorphism of microsatellite repeat fragments are positively correlated with its mutation rate^17^. The GSM47 showed the highest polymorphism in our study may have a higher probability of mutation than other loci, which might be another reason for the mismatches. Therefore, in order to avoid the mistake of excluding paternity caused by STR mutations and PCR error, the basis of excluding paternity in forensic identification can not rely on single locus^18–19^.

An internationally recommended consensus requires at least two mismatched loci between offspring and candidate to exclude paternity^18^. In recent years, a considerable number of laboratories and forensic institutions in China have adopted another consensus, that is, at least three mismatched loci are needed to exclude paternity^19–20^. Microsatellites have decades of forensic experience in human paternity testing. With the widespread use of paternity test kits, it has been found that the mutation rate of microsatellites are higher than previously thought^19–20^. When only one or two exclusion loci occur (usually in a commercial kit with approximately 15 STR loci), the laboratory can add additional loci to adequately exclude the possibility of mutation at the site, or test all candidate fathers to determine whether there is a more matched father. We think that the latter consensus is stricter on the exclusion of paternity, so it may be more appropriate for adoption. In this study all candidate fathers were tested, and the result showed that the two recorded fathers were the highest match among all candidates, thus confirming that the two father's paternity could not be excluded.

In paternity tests with fewer than 14 loci, some candidate fathers achieved a higher level of Trio LOD and Trio Confidence than the recorded fathers (Supplementary Table 4c, 4d and 4e). However, the management records of the zoo could exclude them to be the true fathers, because the candidates and the corresponding recorded mothers were not in the same cage during the mating season before the birth of the offspring or at any time. In these cases, the individuals had the same number of matched loci, but their scores were slightly different. This was caused by the likelihood equations adopted by the software, which had not much practical meaning. In a word, although the two loci had a few mismatches in our study, they did not cause errors in the results of the paternity test. On the contrary, removing any of them will make the result of the paternity test more complicated. Therefore, we recommend the 14 loci combinations for a paternity test in future research and work.

##### Genetic diversity analysis

The genetic diversity of golden snub-nosed monkey in Chengdu Zoo and Pingwu country were analyzed and compared as another application of the marker system. Previous studies have always used different STRs for different population, so there is no reliable way to judge the size of genetic diversity between different populations. In this study, two completely unrelated populations were assessed for genetic diversity with the same set of newly developed STR markers. Interestingly, our study found that the captive population had a higher polymorphism than the Pingwu wild population. We analyzed the pedigree of the captive population in detail and found that although this population was small, its genetic background was complex. This population was composed of individuals from multiple sites of four large geographical populations (Qionglai, Minshan, Shennongjia and Gansu) with their offspring cross-breeding in various ways over the past decades. On the other hand, samples from Pingwu wild population were only collected from individuals from two small groups. Because of habitat fragmentation, their gene exchange with other wild populations was limited resulting in relatively low genetic diversity compared to the captive population. These results suggest that the marker system can effectively analyze genetic diversity, compare genetic differences among populations, and can be used to monitor genetic changes within the population.

In the Chengdu Zoo population, six loci (locus 5, 16, 25, 42, 47 and 69) deviated from Hardy–Weinberg equilibrium (P < 0.05). This might cause by a Wahlund Effect^21^, considering the monkeys in the Chengdu Zoo were a small population and they came from a variety of geographic populations. And in captive population, which is far from the ideal biological population, the failure to meet HWE was usually not a reason to discard a locus^22^.

## Conclusion

In summary, by genome-wide screening for STRs in reference genomic data and comparing it with the 12 resequencing genomes in golden snub-nosed monkey, a total of 14 novel polymorphic tetranucleotide marker were proved to be reliable and valid for non-invasion samples to establish a standardized marker system. A subset of seven STR loci was appropriate for individual identification of both Chengdu Zoo captive population and Pingwu wild monkeys. The full set of 14 STR loci was appropriate for paternity assignment and genetic diversity analysis. This marker system showed a remarkably high success rate and a low error rate in the application of fecal samples. The novel system obtained here will facilitate the genetic management for the captive populations, and provide feasible solutions for the long-term assessment of genetic diversity and the formulation of conservation strategies for wild populations.

## Material and methods

### Sample collection

Thirty-four specimens were collected from Chengdu Zoo (22 fecal samples, 6 blood samples and 6 tissue samples), which represented 25 individuals in total. Four individuals were sampled for both fecal and blood/tissue samples, which could be used to test the stability of the markers. All samples were carefully collected to avoid cross-contamination. They were separately stored in sterile bags or EDTA anticoagulation tubes and frozen at – 80 ℃. The pedigree records from Chengdu Zoo indicated that the genetic background of these monkeys is complex. The ancestors were captured from multiple sites of Gansu moutains, Sichuan Qionglai moutains, Sichuan Minshan moutains and Hubei Shennongjia moutains. All samples were used in identification tests and to verify the pedigree records.

Another 30 fecal samples were collected from Pingwu country, Sichuan Province, which mainly came from a small wild family of 18 individuals and a nearby all-male band. These monkeys were attracted by human feeding in 2017, so their pedigree records were incomplete and some kinships needed confirmation. The fecal samples were used to determine the identity and paternity of these monkeys, as well as the genetic diversity of this population. Eight of the samples were acurrately assigned to known individuals, because they were collected immediately after individual defecation was observed. The rest, 22 samples, were collected randomly from multiple locations within a week without any background information. In order to avoid repeated sampling of the same individual, we tried not to take multiple samples in one place, and each sample was collected at a certain interval.

### Genome sequences and STR identification

The assembled genome of golden snub-nosed monkey (NCBI accession: GCA_000769185.1) was analyzed by Krait^23^. Search mode was set to search for perfect STRs. The minimum repeat number from mono- to hexa-nucleotide were set to 12, 7, 5, 4, 4 and 4, respectively. The flank sequence was set to 100 bp. Other parameters were set to default. The repetitive units with circular permutations and on the complementary chains were treated as the same repetition. For example, the AGT stands for AGT, GTA, TAG, TCA, CAT and ATC.

Taking the genomic data as reference, LobSTR 4.0.6^24^ was used to screen for polymorphic tetranucleotide in the 12 resequencing genomes^25^ (Supplementary Table 1). For the first, a LobSTR reference index was constructed based on golden snub-nosed monkey’s STR data (generated as previously described) using a lobstr_index.py script. Then the resequencing genomes were aligned to the reference genome of the golden snub-nosed monkey by default parameters. SAMtools^26^ was used to sort the output BAM files. Finally, we analyzed the allele genotypes of STRs in golden snub-nosed monkeys based on the alignment file of 12 samples. The VCFtools^27^ were used to screen polymorphic tetranucleotide that shared among all the 12 resequencing genomes, by the application of “-min-alleles 3” and “-maf 0.1” parameters.

### DNA extraction and polymorphism STRs amplification

Genomic DNA was extracted using TIANamp Stool DNA Kit and TIANamp Genomic DNA Kit (TIANGEN BIOTECH, Beijing) for fecal samples and blood/tissue samples, respectively. The 20 μl PCR reaction system included 10 μl Mix (2 × Rapid Taq Master Mix, P222-AA, VAZYME BIOTECH, Najing), 8 μl dd H_2_O, 0.5 μl each primer (25uM solution) and 0.5 ~ 1 μl template DNA (about 2 U). The amplification protocols were carried out as follows: initial denaturation 3 min at 95 ℃, 40 cycles (15 s at 95 ℃, 15 s at 51 ℃, 15 s at 72 ℃), final elongation of 3 min at 72℃. PCR products were visualized on a 3% agarose gel and further capillary electrophoresis on ABI 3,100 genetic analyzer. Genotyping data were obtained by GeneMapper 4.0 (Sangon Biotech, Shanghai; and Tsingke Biological Technology, Beijing).

### Development of STR markers

Polymorphic STRs were extracted from the program output and primer sets were designed according to their flanking regions by Primer Premier 6.0 (https://www.premierbiosoft.com/). We compared the published STR sequences for the golden snub-nosed monkey in NCBI with the output sequences of our program to ensure that our STR sequences did not repeat any published sequences. The length of primers designed in the present study ranged from 18 to 22 bp, the annealing temperature was set to around 51℃, and the expected size of products ranged from 100 to 300 bp. We designed similar annealing temperatures for all the primers, so the amplification conditions of STR loci are basically the same. We did this to make the operation of the system easier to share and extend to other researchers and less professional staff.

In-silico PCR (https://genome.ucsc.edu/cgi-bin/hgPcr) was used to predict the size of the product based on the genomic data (Oct. 2014 Rrox_v1/rhiRox1) and whether there was only a single PCR product. Then, 15 samples (5 blood, 6 feces and 4 tissues) were used to test the repeatable amplification by using 51 ℃ annealing temperature under standard PCR conditions. For primer pairs with inefficient or failed amplification, we tested whether adding more mix, template DNA or adjusting annealing temperature could increase the amplification rate. The results showed that doubling the DNA template and/or increasing 0.5 µl of PCR mix was usually enough to amplify the samples which failed in the first amplification.

We synthesized 42 of the 69 primer pairs and used them to amplify target STRs in DNA of both blood/tissue (n = 8) and fecal (n = 7) samples. After amplification, 31 primer pairs showed a single band of the expected product with fluorescent signals on their forward primer 5′ terminal (FAM or HEX). These were tested for polymorphism by using all of 34 samples collected from Chengdu Zoo. If these primers in the tests, especially in fecal sample tests, showed that (1) the success rate was less than 70% (n = 3), (2) a lack of polymorphism (n = 2), (3) more than 30% of the samples were multiple peaks (n = 8), and (4) not an integer multiple of 4 (n = 2), they were excluded from subsequent trials. In the end, 16 primer pairs had polymorphism (allele 2–6) in their loci and amplified well (100%) on both fecal and blood/tissue DNA.

Cervus 3.0.7 was used to check for possible null alleles. All individuals of our samples were included in this test (47 in total). F(Null) frequency of all 16 loci were calculated. In the absence of a null allele, the estimated frequency will be close to zero, and maybe slightly negative. If a locus showed a large positive frequency, it may imply that a null allele is present or an excess of homozygotes.

Since the genome of the golden snub-nosed monkey was assembled only to scaffold and had not yet been annotated to the chromosome, it was not clear whether the 16 novel tetranucleotide STRs were on different chromosomes. However, we BLAST the sequences of 16 loci in GenBank and aligned them with homologous sequences of human and other primates in order to roughly analyze their chromosomal location. The results showed that GSM 31 and 32 were probably linked, due to their homologous sequences of human were both located on chromosome 17. We also used Genepop to test the linkage disequilibrium of these loci, and the results also showed GSM 31 and 32 were likely linked. Beyond that, no significant linkage disequilibrium between loci was found. Therefore, only one of the locus 31 and 32 should be included in the STR system.

To make sure that the same allele calls would be made by different researchers and laboratories in the future, our genotyping was repeated and entrusted to two independent sequencing companies for genotyping. In our study, every PCR included a control sample (ID: XX, Chengdu Zoo). In genotyping analysis, this control sample was used to ensure standardized allele calling between machine runs. These steps were our efforts to avoid different genotyping data for the same locus due to machine differences.

### Sex identification

Unfortunately, our attempts to identify sex of the golden snub-nosed monkeys based on the primer pairs of DEAD-box^28^ which had been used to identify the sex of primates successfully in the previous studies, all failed. Therefore, based on the genomic data, we designed a new primer pair target ZFX and ZFY gene to determine the sex of Golden snub-nosed monkey (Table 1). The PCR reaction system and amplification protocol were the same as for our STRs. PCR products were visualized on a 3% agarose gel. Males were identified by two bands (211 bp and 140 bp) while females were identified by one single band (211 bp). DNA from 25 monkey samples (fecal = 13, blood = 6 and tissue = 6) of known sex (male = 10, female = 15) were first used to test the success rate and accuracy rate of the primer pair. After the 25 samples were easily and correctly identified, all our 64 samples were tested and identified. For duplicate samples (one individual sampled multiple times) in genotyping, their genders were checked to ensure that they were of the same gender.

### Verification of successful amplification on fecal samples

The 22 fecal DNA from captive population were used to test whether polymorphic STR markers were highly sensitive to and could be applied to fecal DNA. Each marker was tested three times on each sample. The markers would be excluded if (1) the amplification success rate was less than 70%; (2) More than 30% of the samples showed multiple peaks in the genotyping results, and the stutter peaks appeared in more than two repeated tests.

### Verification of stability of these polymorphic STRs

Four individuals were sampled for both fecal and blood/tissue samples. The results of genotyping in blood and feces of the same individual (4 in total) were compared to evaluate the reliability and stability of STR markers. The markers would be excluded if the genotyping results of blood and fecal samples were inconsistent. In addition to test whether these STRs can be used to the fecal samples that were exposure to the wild environment, thirty fecal samples from the wild (1–7 days of wild exposure) were also used to test the amplification success rate and accuracy of these STRs.

### The application of the novel STR marker system

#### Individual identification

Firstly, by using all 16 markers we identified 47 individuals in our 64 samples. Then the genotypes of the 47 individuals (25 from Chengdu Zoo and 22 from Pingwu) were used to establish a dataset. Cervus 3.0.7 (https://www.fieldgenetics.com) were used to analyze the Total H_O_, H_E_ and PIC of this dataset. And then we tried three methods to arrange these markers, according to their H_O_, H_E_ and PIC value from high to low respectively. The markers with the highest value were select one by one, and its PID and PIDsib values were calculated. When the accumulated PID and PIDsib reached less than 0.001 and 0.01 respectively, the minimum number of loci needed for individual recognition was obtained.

After comparing these three methods, we found the best method was based on H_E_ value. According to the STRs ordered by H_E_ value, we initially found that eight markers were needed to reach the thresholds of PID and PIDsib. However, we further found that GSM25 is not necessary for this individual identification system. When the markers reached seven, only two individuals (PW14 and PWA) were exact match. And they had the same allele on GSM25, but different allele on GSM75. If GSM25 is replaced by GSM75, the seven markers can also reach the threshold and discriminate individuals. Therefore, we ended up with seven markers that can effectively identify individual.

#### Paternity test

In addition, the novel STR markers were used to determine the paternity both in this captive and the wild populations. Cervus 3.0.7 were used to conduct offspring analysis. For each monkey in captivity, the genotype of the recorded father was compared with the genotypes of all potential sire candidates to verify the lineage record. In the wild population, all male samples, including the dominant male, were selected as candidate father to determine which one was the biological father of a 1-year old cub who lacked pedigree records.

#### Statistical and genetic data analysis

We also analyzed the genetic diversity of golden snub-nosed monkey population both in captive and in wild. The observed heterozygosity (H_O_), expected heterozygosity (H_E_), polymorphic information content (PIC), individual identification (PID and PIDsib) and paternity testing were calculated by Cervus 3.0.7 (https://www.fieldgenetics.com)^10^. Hardy–Weinberg equilibrium (HWE P-value) were calculated by Genepop 4.2 (https://www.genepop.curtin.edu.au)^29^.

### Ethical approval

All samples were collected in accordance with the regulations on the implementation of China’s terrestrial wildlife protection (order [2016] No. 666 of the State Council). The ethical review of this study was approved by the ethics committee of the College of life sciences, Sichuan University.

## Supplementary information

Supplementary file1 (DOCX 15 kb)

Supplementary file2 (DOCX 12 kb)

Supplementary file3 (XLSX 15 kb)

Supplementary file4 (DOCX 55 kb)

Supplementary file5 (DOCX 16 kb)

Supplementary file6 (DOC 29 kb)

## Data Availability

All data generated or analysed during this study are included in this published article (and its Supplementary Information files).

## References

[1] Wang S, Xie Y (2004). China Species Red List.

[2] Yongcheng, L. & Richardson, M. *Rhinopithecus roxellana*. The IUCN Red List of Threatened Species 2008: e.T19596A8985735. (2008).

[3] Yu Z, Xia Q, Fan X (2018). Fertility and management of a captive population of golden monkey. Chin. J. Wildl..

[4] Pan D (2005). Microsatellite polymorphisms of 1 Sichuan golden monkeys. Chin. Sci. Bull..

[5] Chang ZF, Liu ZJ, Yang JY, Li M, Vigilant L (2012). Noninvasive genetic assessment of the population trend and sex ratio of the Shennongjia population of Sichuan snub-nosed monkeys (*Rhinopithecus roxellana*). Chin. Sci. Bull..

[6] Zhou Y (2018). Genetic structure of the golden snub-nosed monkey in Shennongjia National Natural Reserve based on microsatellite DNA markers. Acta Ecol. Sin..

[7] He L, Zhang Y, Peng H, Li D, Li D (2010). Genetic diversity of *Rhinopithecus roxellana* in Shennongjia National Nature Reserve as estimated by non-invasive DNA technology. Acta Ecol. Sin..

[8] Chang ZF (2012). Human influence on the population decline and loss of genetic diversity in a small and isolated population of sichuan snub-nosed monkeys (*Rhinopithecus roxellana*). Genetica.

[9] Fang HX (2010). Animal species and population size in Chinese zoos. Chin. J. Zool..

[10] Kalinowski ST, Taper ML, Marshall TC (2007). Revising how the computer program CERVUS accommodates genotyping error increases success in paternity assignment. Mol. Ecol..

[11] Duan YM, Huang K, Qi XG, Li BG (2017). Characterization of 29 highly polymorphic microsatellite markers for golden snub-nosed monkey (*Rhinopithecus roxellana*). Conserv. Genet. Resour..

[12] Pompanon F, Bonin A, Bellemain E, Taberlet P (2005). Genotyping errors: Causes, consequences and solutions. Nat. Rev. Genet..

[13] Taberlet P (1996). Reliable genotyping of samples with very low DNA quantities using PCR. Nucl. Acid Res..

[14] Huang J (2015). Genome-wide survey and analysis of microsatellites in giant panda (*Ailuropoda melanoleuca*), with a focus on the applications of a novel microsatellite marker system. BMC Genomics.

[15] Labuschagne C, Nupen L, Kotzé A, Grobler PJ, Dalton DL (2015). Assessment of microsatellite and SNP markers for parentage assignment in ex situ African Penguin (*Spheniscus demersus*) populations. Ecol. Evol..

[16] Coetzer WG, Downs CT, Perrin MR, Willows-Munro S (2017). Testing of microsatellite multiplexes for individual identification of cape parrots (*Poicephalus robustus*): paternity testing and monitoring trade. PeerJ.

[17] Ellegren H (2000). Microsatellite mutations in the germline: Implications for evolutionary inference. Trends Genet..

[18] Jones AG, Small CM, Paczolt KA, Ratterman NL (2010). A practical guide to methods of parentage analysis. Mol. Ecol. Resour..

[19] Jiang HJ, Yin L, Mu HF, Chen F, He NY, Du Z (2017). Feasibility study of STR typing based on next-generation sequencing. Chin. J. Forensic Med..

[20] Fang JX, Cheng DL (2002). Evaluation on the number and value of STR loci applied in paternity identification. J. Forensic Med..

[21] Johnson MS, Black R (1984). The Wahlund effect and the geographical scale of variation in the intertidal limpet *Siphonaria *sp. Mar. Biol..

[22] Selkoe KA, Toonen RJ (2006). Microsatellites for ecologists: A practical guide to using and evaluating microsatellite markers. Ecol. Lett..

[23] Du L, Zhang C, Liu Q, Zhang X, Yue B (2017). Krait: An ultrafast tool for genome-wide survey of microsatellites and primer design. Bioinformatics.

[24] Gymrek M, Golan D, Rosset S, Erlich Y (2012). LobSTR: A short tandem repeat profiler for personal genomes. Genome Res..

[25] Zhou X (2016). Population genomics reveals low genetic diversity and adaptation to hypoxia in snub-nosed monkeys. Mol. Biol. Evol..

[26] Li H (2009). The Sequence alignment/map (SAM) format and SAMtools. Bioinformatics.

[27] Danecek P (2011). The variant call format and VCFtools. Bioinformatics.

[28] Villesen P, Fredsted T (2006). A new sex identification tool: One primer pair can reliably sex ape and monkey DNA samples. Conserv. Genet..

[29] Rousset F (2008). Genepop'007: A complete re-implementation of the Genepop software for Windows and Linux. Mol. Ecol. Resour..

