# Supplementary material for: Genome-wide screening of microsatellites in golden snub-nosed monkey (Rhinopithecus roxellana), for the development of a standardized genetic marker system
Source: Sci Rep. 2020 Jun 30;10:10614. doi: 10.1038/s41598-020-67451-2 (PMC7326997; doi:10.1038/s41598-020-67451-2)
Supplement: Supplementary file 1 — Supplementary file1 (DOCX 15 kb) [file 41598_2020_67451_MOESM1_ESM.docx]

**Supplementary Table 1.** The 12 resequencing genomes used to develop the standardized STR system.

| Population | NCBI BioSample name | Size |
| --- | --- | --- |
| Gan Su | R.rox_GS-2011A047 | 25.2Gb |
|  | R.rox_GS-2011A049 | 19.3Gb |
|  | R.rox_GS-2011A052 | 57.7Gb |
|  |  |  |
| Min Shan | R.rox_MS-2011A003 | 18.7Gb |
|  | R.rox_MS-2011A007 | 21.7Gb |
|  | R.rox_MS-2011A041 | 19.2Gb |
|  |  |  |
| Qiong Lai | R.rox_QLA-2012A005 | 23.8Gb |
|  | R.rox_QLA-2012A007 | 19.1Gb |
|  | R.rox_QLA-2013A007 | 19.6Gb |
|  |  |  |
| Shen Nong Jia | R.rox_SNJ-2013A008 | 24.3Gb |
|  | R.rox_SNJ-2013A009 | 26.1Gb |
|  | R.rox_SNJ-2013A022 | 23.1Gb |
