# Supplementary material for: Genome-wide screening of microsatellites in golden snub-nosed monkey (Rhinopithecus roxellana), for the development of a standardized genetic marker system
Source: Sci Rep. 2020 Jun 30;10:10614. doi: 10.1038/s41598-020-67451-2 (PMC7326997; doi:10.1038/s41598-020-67451-2)
Supplement: Supplementary file 2 — Supplementary file2 (DOCX 12 kb) [file 41598_2020_67451_MOESM2_ESM.docx]

Supplementary Table 2. The tests on the relationship between the exposure time of fecal samples and the stability of the 16 loci.

| Exposure time | 1day | 3day | 4day | 5day | 6~7day |
| --- | --- | --- | --- | --- | --- |
| Number of fecal samples | 7 | 4 | 5 | 8 | 6 |
| Success rate on PCR amplification | 100% | 100% | 100% | 100% | 100% |
