# Supplementary material for: Genome-wide screening of microsatellites in golden snub-nosed monkey (Rhinopithecus roxellana), for the development of a standardized genetic marker system
Source: Sci Rep. 2020 Jun 30;10:10614. doi: 10.1038/s41598-020-67451-2 (PMC7326997; doi:10.1038/s41598-020-67451-2)
Supplement: Supplementary file 4 — Supplementary file4 (DOCX 55 kb) [file 41598_2020_67451_MOESM4_ESM.docx]

Supplementary Table 4a. Paternity test with 16 loci.

| Offspring ID | Loci Typed | Candidate Mother ID | Population From | Pair Loci Mismatching | Candidate Father ID | Population From | Pair Loci Mismatching | Trio Loci Mismatching | Trio LOD Score | Trio Confidence |
| --- | --- | --- | --- | --- | --- | --- | --- | --- | --- | --- |
| G1 ♀ | 16 | G2 | Shennongjia | 0 | A180 | Minshan Shehong | 2 | 6 | -1.53E+01 |  |
| G1 ♀ | 16 | G2 |  | 0 | A230 | No record | 3 | 7 | -1.88E+01 |  |
| G1 ♀ | 16 | G2 |  | 0 | B27 | No record | 2 | 2 | 2.50E+00 |  |
| G1 ♀ | 16 | G2 |  | 0 | CC | Chengdu zoo | 0 | 0 | 1.24E+01 | ***** |
| G1 ♀ | 16 | G2 |  | 0 | C16 | Qionglai Longchi | 4 | 7 | -1.99E+01 |  |
| G1 ♀ | 16 | G2 |  | 0 | SW3 | Minshan Beichuan | 3 | 7 | -2.01E+01 |  |
| G1 ♀ | 16 | G2 |  | 0 | W2 | Qionglai Heishuihe | 7 | 9 | -2.84E+01 |  |
| G1 ♀ | 16 | G2 |  | 0 | Y3 | Minshan Xindu | 3 | 5 | -9.84E+00 |  |
| G1 ♀ | 16 | G2 |  | 0 | Y5 | Shanghai zoo | 4 | 7 | -2.05E+01 |  |
| G6 ♂ | 16 | J2 | Chengdu zoo | 0 | A180 | Minshan Shehong | 7 | 9 | -3.22E+01 |  |
| G6 ♂ | 16 | J2 |  | 0 | A230 | No record | 1 | 4 | -7.64E+00 |  |
| G6 ♂ | 16 | J2 |  | 0 | B27 | No record | 5 | 8 | -2.90E+01 |  |
| G6 ♂ | 16 | J2 |  | 0 | CC | Chengdu zoo | 0 | 5 | -1.48E+01 |  |
| G6 ♂ | 16 | J2 |  | 0 | C16 | Qionglai Longchi | 0 | 0 | 1.26E+01 | ***** |
| G6 ♂ | 16 | J2 |  | 0 | SW3 | Minshan Beichuan | 4 | 6 | -1.87E+01 |  |
| G6 ♂ | 16 | J2 |  | 0 | W2 | Qionglai Heishuihe | 4 | 6 | -1.58E+01 |  |
| G6 ♂ | 16 | J2 |  | 0 | Y3 | Minshan Xindu | 4 | 8 | -2.68E+01 |  |
| G6 ♂ | 16 | J2 |  | 0 | Y5 | Shanghai zoo | 4 | 8 | -2.60E+01 |  |
| J5 ♂ | 16 | J2 | Chengdu zoo | 1 (GSM47) | A180 | Minshan Shehong | 7 | 8 | -2.73E+01 |  |
| J5 ♂ | 16 | J2 |  | 1 | A230 | No record | 3 | 4 | -7.38E+00 |  |
| J5 ♂ | 16 | J2 |  | 1 | B27 | No record | 3 | 4 | -1.11E+01 |  |
| J5 ♂ | 16 | J2 |  | 1 | CC | Chengdu zoo | 1 (GSM47) | 3 | -4.70E+00 |  |
| J5 ♂ | 16 | J2 |  | 1 | C16 | Qionglai Longchi | 1 (GSM05) | 2 | 3.29E+00 | ***** |
| J5 ♂ | 16 | J2 |  | 1 | SW3 | Minshan Beichuan | 6 | 7 | -2.25E+01 |  |
| J5 ♂ | 16 | J2 |  | 1 | W2 | Qionglai Heishuihe | 6 | 7 | -2.12E+01 |  |
| J5 ♂ | 16 | J2 |  | 1 | Y3 | Minshan Xindu | 4 | 5 | -1.28E+01 |  |
| J5 ♂ | 16 | J2 |  | 1 | Y5 | Shanghai zoo | 4 | 6 | -1.64E+01 |  |
| Z1♀ | 16 | S2 | Chengdu zoo | 1 (GSM05) | A180 | Minshan Shehong | 2 (GSM05, 47) | 2 | -3.75E+00 | ***** |
| Z1♀ | 16 | S2 |  | 1 | A230 | No record | 1 (GSM69) | 3 | -4.36E+00 |  |
| Z1♀ | 16 | S2 |  | 1 | B27 | No record | 3 | 4 | -1.18E+01 |  |
| Z1♀ | 16 | S2 |  | 1 | CC | Chengdu zoo | 1 (GSM69) | 3 | -6.81E+00 |  |
| Z1♀ | 16 | S2 |  | 1 | C16 | Qionglai Longchi | 5 | 6 | -1.92E+01 |  |
| Z1♀ | 16 | S2 |  | 1 | SW3 | Minshan Beichuan | 3 | 4 | -1.01E+01 |  |
| Z1♀ | 16 | S2 |  | 1 | W2 | Qionglai Heishuihe | 4 | 5 | -1.36E+01 |  |
| Z1♀ | 16 | S2 |  | 1 | Y3 | Minshan Xindu | 3 | 5 | -1.50E+01 |  |
| Z1♀ | 16 | S2 |  | 1 | Y5 | Shanghai zoo | 4 | 5 | -1.34E+01 |  |
| Y1♂ | 16 | S1 | Chengdu zoo | 0 | A180 | Minshan Shehong | 7 | 8 | -2.79E+01 |  |
| Y1♂ | 16 | S1 |  | 0 | A230 | No record | 3 | 5 | -1.39E+01 |  |
| Y1♂ | 16 | S1 |  | 0 | B27 | No record | 0 | 1 | 1.83E+00 |  |
| Y1♂ | 16 | S1 |  | 0 | CC | Chengdu zoo | 0 | 0 | 5.34E+00 | ***** |
| Y1♂ | 16 | S1 |  | 0 | C16 | Qionglai Longchi | 5 | 6 | -1.85E+01 |  |
| Y1♂ | 16 | S1 |  | 0 | SW3 | Minshan Beichuan | 6 | 6 | -2.07E+01 |  |
| Y1♂ | 16 | S1 |  | 0 | W2 | Qionglai Heishuihe | 6 | 8 | -2.67E+01 |  |
| Y1♂ | 16 | S1 |  | 0 | Y3 | Minshan Xindu | 1 | 1 | 4.58E+00 |  |
| Y1♂ | 16 | S1 |  | 0 | Y5 | Shanghai zoo | 2 | 6 | -1.77E+01 |  |
| H3♀ | 16 | S2 | Chengdu zoo | 0 | A180 | Minshan Shehong | 0 | 0 | 1.48E+01 | ***** |
| H3♀ | 16 | S2 |  | 0 | A230 | No record | 3 | 8 | -2.61E+01 |  |
| H3♀ | 16 | S2 |  | 0 | B27 | No record | 2 | 6 | -1.74E+01 |  |
| H3♀ | 16 | S2 |  | 0 | CC | Chengdu zoo | 3 | 8 | -2.66E+01 |  |
| H3♀ | 16 | S2 |  | 0 | C16 | Qionglai Longchi | 8 | 10 | -3.63E+01 |  |
| H3♀ | 16 | S2 |  | 0 | SW3 | Minshan Beichuan | 2 | 5 | -1.22E+01 |  |
| H3♀ | 16 | S2 |  | 0 | W2 | Qionglai Heishuihe | 6 | 9 | -2.94E+01 |  |
| H3♀ | 16 | S2 |  | 0 | Y3 | Minshan Xindu | 4 | 8 | -2.49E+01 |  |
| H3♀ | 16 | S2 |  | 0 | Y5 | Shanghai zoo | 5 | 9 | -3.10E+01 |  |
| PW15♀ | 15 | PWB | Minshan Pingwu | 0 | PW14 | Minshan Pingwu | 1 | 1 | -6.08E-01 |  |
| PW15♀ | 15 | PWB |  | 0 | PW16 | Minshan Pingwu | 3 | 4 | -1.20E+01 |  |
| PW15♀ | 15 | PWB |  | 0 | PW19 | Minshan Pingwu | 2 | 4 | -1.16E+01 |  |
| PW15♀ | 15 | PWB |  | 0 | PW3 | Minshan Pingwu | 3 | 4 | -1.40E+01 |  |
| PW15♀ | 15 | PWB |  | 0 | PW7 | Minshan Pingwu | 3 | 4 | -1.27E+01 |  |
| PW15♀ | 15 | PWB |  | 0 | PW9 | Minshan Pingwu | 3 | 4 | -1.41E+01 |  |
| PW15♀ | 15 | PWB |  | 0 | PWA | Minshan Pingwu | 0 | 0 | 2.81E+00 | ***** |
| PW15♀ | 15 | PWB |  | 0 | PWF | Minshan Pingwu | 1 | 2 | -1.66E+00 |  |
| PW15♀ | 15 | PWB |  | 0 | PWG | Minshan Pingwu | 2 | 3 | -1.11E+01 |  |

Trio confidence: * for strict confidence, + for relaxed confidence, and - for a most likely candidate parent not assigned parentage.

Supplementary Table 4b. Paternity test with 14 loci.

| Offspring ID | Loci Typed | Candidate Mother ID | Population From | Pair Loci Mismatching | Candidate Father ID | Population From | Pair Loci Mismatching | Trio Loci Mismatching | Trio LOD Score | Trio Confidence |
| --- | --- | --- | --- | --- | --- | --- | --- | --- | --- | --- |
| G1 ♀ | 14 | G2 | Shennongjia | 0 | A180 | Minshan Shehong | 2 | 6 | -1.50E+01 |  |
| G1 ♀ | 14 | G2 |  | 0 | A230 | No record | 3 | 7 | -1.91E+01 |  |
| G1 ♀ | 14 | G2 |  | 0 | B27 | No record | 2 | 2 | 2.83E+00 |  |
| G1 ♀ | 14 | G2 |  | 0 | CC | Chengdu zoo | 0 | 0 | 1.20E+01 | **-** |
| G1 ♀ | 14 | G2 |  | 0 | C16 | Qionglai Longchi | 4 | 7 | -1.95E+01 |  |
| G1 ♀ | 14 | G2 |  | 0 | SW3 | Minshan Beichuan | 3 | 7 | -2.04E+01 |  |
| G1 ♀ | 14 | G2 |  | 0 | W2 | Qionglai Heishuihe | 7 | 9 | -2.80E+01 |  |
| G1 ♀ | 14 | G2 |  | 0 | Y3 | Minshan Xindu | 3 | 5 | -1.02E+01 |  |
| G1 ♀ | 14 | G2 |  | 0 | Y5 | Shanghai zoo | 4 | 7 | -2.08E+01 |  |
| G6 ♂ | 14 | J2 | Chengdu zoo | 0 | A180 | Minshan Shehong | 7 | 7 | -2.46E+01 |  |
| G6 ♂ | 14 | J2 |  | 0 | A230 | No record | 1 | 2 | -2.22E-01 |  |
| G6 ♂ | 14 | J2 |  | 0 | B27 | No record | 5 | 8 | -2.99E+01 |  |
| G6 ♂ | 14 | J2 |  | 0 | CC | Chengdu zoo | 0 | 4 | -1.10E+01 |  |
| G6 ♂ | 14 | J2 |  | 0 | C16 | Qionglai Longchi | 0 | 0 | 1.10E+01 | **-** |
| G6 ♂ | 14 | J2 |  | 0 | SW3 | Minshan Beichuan | 4 | 4 | -1.13E+01 |  |
| G6 ♂ | 14 | J2 |  | 0 | W2 | Qionglai Heishuihe | 4 | 5 | -1.31E+01 |  |
| G6 ♂ | 14 | J2 |  | 0 | Y3 | Minshan Xindu | 4 | 7 | -2.37E+01 |  |
| G6 ♂ | 14 | J2 |  | 0 | Y5 | Shanghai zoo | 4 | 7 | -2.22E+01 |  |
| J5 ♂ | 14 | J2 | Chengdu zoo | 1 (GSM47) | A180 | Minshan Shehong | 7 | 7 | -2.38E+01 |  |
| J5 ♂ | 14 | J2 |  | 1 | A230 | No record | 3 | 3 | -4.07E+00 |  |
| J5 ♂ | 14 | J2 |  | 1 | B27 | No record | 3 | 4 | -1.08E+01 |  |
| J5 ♂ | 14 | J2 |  | 1 | CC | Chengdu zoo | 1 | 3 | -5.04E+00 |  |
| J5 ♂ | 14 | J2 |  | 1 | C16 | Qionglai Longchi | 1 | 2 | 2.94E+00 | **-** |
| J5 ♂ | 14 | J2 |  | 1 | SW3 | Minshan Beichuan | 6 | 6 | -1.92E+01 |  |
| J5 ♂ | 14 | J2 |  | 1 | W2 | Qionglai Heishuihe | 6 | 6 | -1.72E+01 |  |
| J5 ♂ | 14 | J2 |  | 1 | Y3 | Minshan Xindu | 4 | 5 | -1.38E+01 |  |
| J5 ♂ | 14 | J2 |  | 1 | Y5 | Shanghai zoo | 4 | 6 | -1.68E+01 |  |
| Z1♀ | 14 | S2 | Chengdu zoo | 1 (GSM05) | A180 | Minshan Shehong | 2 | 2 | -3.42E+00 | **-** |
| Z1♀ | 14 | S2 |  | 1 | A230 | No record | 1 | 3 | -4.71E+00 |  |
| Z1♀ | 14 | S2 |  | 1 | B27 | No record | 3 | 4 | -1.15E+01 |  |
| Z1♀ | 14 | S2 |  | 1 | CC | Chengdu zoo | 1 | 3 | -7.16E+00 |  |
| Z1♀ | 14 | S2 |  | 1 | C16 | Qionglai Longchi | 5 | 6 | -1.89E+01 |  |
| Z1♀ | 14 | S2 |  | 1 | SW3 | Minshan Beichuan | 3 | 4 | -1.04E+01 |  |
| Z1♀ | 14 | S2 |  | 1 | W2 | Qionglai Heishuihe | 4 | 5 | -1.33E+01 |  |
| Z1♀ | 14 | S2 |  | 1 | Y3 | Minshan Xindu | 3 | 5 | -1.53E+01 |  |
| Z1♀ | 14 | S2 |  | 1 | Y5 | Shanghai zoo | 4 | 5 | -1.38E+01 |  |
| Y1♂ | 14 | S1 | Chengdu zoo | 0 | A180 | Minshan Shehong | 6 | 7 | -2.43E+01 |  |
| Y1♂ | 14 | S1 |  | 0 | A230 | No record | 2 | 4 | -1.03E+01 |  |
| Y1♂ | 14 | S1 |  | 0 | B27 | No record | 0 | 1 | 1.91E+00 |  |
| Y1♂ | 14 | S1 |  | 0 | CC | Chengdu zoo | 0 | 0 | 4.74E+00 | **-** |
| Y1♂ | 14 | S1 |  | 0 | C16 | Qionglai Longchi | 5 | 6 | -1.91E+01 |  |
| Y1♂ | 14 | S1 |  | 0 | SW3 | Minshan Beichuan | 5 | 5 | -1.71E+01 |  |
| Y1♂ | 14 | S1 |  | 0 | W2 | Qionglai Heishuihe | 5 | 7 | -2.25E+01 |  |
| Y1♂ | 14 | S1 |  | 0 | Y3 | Minshan Xindu | 1 | 1 | 3.30E+00 |  |
| Y1♂ | 14 | S1 |  | 0 | Y5 | Shanghai zoo | 2 | 6 | -1.83E+01 |  |
| H3♀ | 14 | S2 | Chengdu zoo | 0 | A180 | Minshan Shehong | 0 | 0 | 1.28E+01 | **-** |
| H3♀ | 14 | S2 |  | 0 | A230 | No record | 3 | 7 | -2.26E+01 |  |
| H3♀ | 14 | S2 |  | 0 | B27 | No record | 2 | 5 | -1.30E+01 |  |
| H3♀ | 14 | S2 |  | 0 | CC | Chengdu zoo | 3 | 7 | -2.29E+01 |  |
| H3♀ | 14 | S2 |  | 0 | C16 | Qionglai Longchi | 7 | 9 | -3.16E+01 |  |
| H3♀ | 14 | S2 |  | 0 | SW3 | Minshan Beichuan | 2 | 4 | -8.74E+00 |  |
| H3♀ | 14 | S2 |  | 0 | W2 | Qionglai Heishuihe | 6 | 8 | -2.52E+01 |  |
| H3♀ | 14 | S2 |  | 0 | Y3 | Minshan Xindu | 3 | 7 | -2.09E+01 |  |
| H3♀ | 14 | S2 |  | 0 | Y5 | Shanghai zoo | 5 | 8 | -2.73E+01 |  |
| PW15♀ | 14 | PWB | Minshan Pingwu | 0 | PW14 | Minshan Pingwu | 1 | 1 | -8.67E-01 |  |
| PW15♀ | 14 | PWB |  | 0 | PW16 | Minshan Pingwu | 3 | 3 | -8.38E+00 |  |
| PW15♀ | 14 | PWB |  | 0 | PW19 | Minshan Pingwu | 2 | 3 | -7.64E+00 |  |
| PW15♀ | 14 | PWB |  | 0 | PW3 | Minshan Pingwu | 3 | 3 | -1.04E+01 |  |
| PW15♀ | 14 | PWB |  | 0 | PW7 | Minshan Pingwu | 3 | 3 | -9.06E+00 |  |
| PW15♀ | 14 | PWB |  | 0 | PW9 | Minshan Pingwu | 3 | 3 | -1.04E+01 |  |
| PW15♀ | 14 | PWB |  | 0 | PWA | Minshan Pingwu | 0 | 0 | 2.54E+00 | **-** |
| PW15♀ | 14 | PWB |  | 0 | PWF | Minshan Pingwu | 1 | 1 | 2.39E+00 |  |
| PW15♀ | 14 | PWB |  | 0 | PWG | Minshan Pingwu | 2 | 2 | -7.36E+00 |  |

Trio confidence: * for strict confidence, + for relaxed confidence, and - for a most likely candidate parent not assigned parentage.

Supplementary Table 4c. Paternity test with 13 loci.

| Offspring ID | Loci Typed | Candidate Mother ID | Population From | Pair Loci Mismatching | Candidate Father ID | Population From | Pair Loci Mismatching | Trio Loci Mismatching | Trio LOD Score | Trio Confidence |
| --- | --- | --- | --- | --- | --- | --- | --- | --- | --- | --- |
| G1 ♀ | 13 | G2 | Shennongjia | 0 | A180 | Minshan Shehong | 2 | 6 | -1.63E+01 |  |
| G1 ♀ | 13 | G2 |  | 0 | A230 | No record | 2 | 6 | -1.56E+01 |  |
| G1 ♀ | 13 | G2 |  | 0 | B27 | No record | 2 | 2 | 1.54E+00 |  |
| G1 ♀ | 13 | G2 |  | 0 | CC | Chengdu zoo | 0 | 0 | 1.14E+01 | **-** |
| G1 ♀ | 13 | G2 |  | 0 | C16 | Qionglai Longchi | 3 | 6 | -1.60E+01 |  |
| G1 ♀ | 13 | G2 |  | 0 | SW3 | Minshan Beichuan | 3 | 7 | -2.10E+01 |  |
| G1 ♀ | 13 | G2 |  | 0 | W2 | Qionglai Heishuihe | 6 | 8 | -2.45E+01 |  |
| G1 ♀ | 13 | G2 |  | 0 | Y3 | Minshan Xindu | 3 | 5 | -1.08E+01 |  |
| G1 ♀ | 13 | G2 |  | 0 | Y5 | Shanghai zoo | 3 | 6 | -1.73E+01 |  |
| G6 ♂ | 13 | J2 | Chengdu zoo | 0 | A180 | Minshan Shehong | 6 | 6 | -2.07E+01 |  |
| G6 ♂ | 13 | J2 |  | 0 | A230 | No record | 1 | 2 | -9.85E-01 |  |
| G6 ♂ | 13 | J2 |  | 0 | B27 | No record | 4 | 7 | -2.60E+01 |  |
| G6 ♂ | 13 | J2 |  | 0 | CC | Chengdu zoo | 0 | 4 | -1.11E+01 |  |
| G6 ♂ | 13 | J2 |  | 0 | C16 | Qionglai Longchi | 0 | 0 | 1.02E+01 | **-** |
| G6 ♂ | 13 | J2 |  | 0 | SW3 | Minshan Beichuan | 4 | 4 | -1.14E+01 |  |
| G6 ♂ | 13 | J2 |  | 0 | W2 | Qionglai Heishuihe | 4 | 5 | -1.39E+01 |  |
| G6 ♂ | 13 | J2 |  | 0 | Y3 | Minshan Xindu | 4 | 7 | -2.38E+01 |  |
| G6 ♂ | 13 | J2 |  | 0 | Y5 | Shanghai zoo | 4 | 7 | -2.30E+01 |  |
| J5 ♂ | 13 | J2 | Chengdu zoo | 1 (GSM47) | A180 | Minshan Shehong | 7 | 7 | -2.44E+01 |  |
| J5 ♂ | 13 | J2 |  | 1 | A230 | No record | 2 | 2 | -1.32E-01 |  |
| J5 ♂ | 13 | J2 |  | 1 | B27 | No record | 3 | 4 | -1.14E+01 |  |
| J5 ♂ | 13 | J2 |  | 1 | CC | Chengdu zoo | 1 | 3 | -4.97E+00 |  |
| J5 ♂ | 13 | J2 |  | 1 | C16 | Qionglai Longchi | 0 | 1 | 6.87E+00 | **-** |
| J5 ♂ | 13 | J2 |  | 1 | SW3 | Minshan Beichuan | 6 | 6 | -1.91E+01 |  |
| J5 ♂ | 13 | J2 |  | 1 | W2 | Qionglai Heishuihe | 5 | 5 | -1.33E+01 |  |
| J5 ♂ | 13 | J2 |  | 1 | Y3 | Minshan Xindu | 4 | 5 | -1.37E+01 |  |
| J5 ♂ | 13 | J2 |  | 1 | Y5 | Shanghai zoo | 3 | 5 | -1.28E+01 |  |
| Z1♀ | 13 | S2 | Chengdu zoo | 0 | A180 | Minshan Shehong | 1 | 1 | 1.18E+00 | **-** |
| Z1♀ | 13 | S2 |  | 0 | A230 | No record | 1 | 2 | -1.22E+00 |  |
| Z1♀ | 13 | S2 |  | 0 | B27 | No record | 2 | 3 | -6.89E+00 |  |
| Z1♀ | 13 | S2 |  | 0 | CC | Chengdu zoo | 1 | 2 | -3.26E+00 |  |
| Z1♀ | 13 | S2 |  | 0 | C16 | Qionglai Longchi | 5 | 5 | -1.54E+01 |  |
| Z1♀ | 13 | S2 |  | 0 | SW3 | Minshan Beichuan | 3 | 3 | -6.50E+00 |  |
| Z1♀ | 13 | S2 |  | 0 | W2 | Qionglai Heishuihe | 4 | 4 | -9.77E+00 |  |
| Z1♀ | 13 | S2 |  | 0 | Y3 | Minshan Xindu | 3 | 4 | -1.14E+01 |  |
| Z1♀ | 13 | S2 |  | 0 | Y5 | Shanghai zoo | 4 | 4 | -1.03E+01 |  |
| Y1♂ | 13 | S1 | Chengdu zoo | 0 | A180 | Minshan Shehong | 6 | 6 | -2.08E+01 |  |
| Y1♂ | 13 | S1 |  | 0 | A230 | No record | 2 | 4 | -1.10E+01 |  |
| Y1♂ | 13 | S1 |  | 0 | B27 | No record | 0 | 0 | 5.46E+00 | - (error) |
| Y1♂ | 13 | S1 |  | 0 | CC | Chengdu zoo | 0 | 0 | 4.74E+00 | (R F) |
| Y1♂ | 13 | S1 |  | 0 | C16 | Qionglai Longchi | 5 | 6 | -1.98E+01 |  |
| Y1♂ | 13 | S1 |  | 0 | SW3 | Minshan Beichuan | 5 | 5 | -1.71E+01 |  |
| Y1♂ | 13 | S1 |  | 0 | W2 | Qionglai Heishuihe | 5 | 7 | -2.31E+01 |  |
| Y1♂ | 13 | S1 |  | 0 | Y3 | Minshan Xindu | 1 | 1 | 3.30E+00 |  |
| Y1♂ | 13 | S1 |  | 0 | Y5 | Shanghai zoo | 2 | 6 | -1.90E+01 |  |
| H3♀ | 13 | S2 | Chengdu zoo | 0 | A180 | Minshan Shehong | 0 | 0 | 1.15E+01 | **-** |
| H3♀ | 13 | S2 |  | 0 | A230 | No record | 2 | 6 | -1.91E+01 |  |
| H3♀ | 13 | S2 |  | 0 | B27 | No record | 2 | 5 | -1.43E+01 |  |
| H3♀ | 13 | S2 |  | 0 | CC | Chengdu zoo | 3 | 7 | -2.35E+01 |  |
| H3♀ | 13 | S2 |  | 0 | C16 | Qionglai Longchi | 6 | 8 | -2.81E+01 |  |
| H3♀ | 13 | S2 |  | 0 | SW3 | Minshan Beichuan | 2 | 4 | -9.34E+00 |  |
| H3♀ | 13 | S2 |  | 0 | W2 | Qionglai Heishuihe | 5 | 7 | -2.16E+01 |  |
| H3♀ | 13 | S2 |  | 0 | Y3 | Minshan Xindu | 3 | 7 | -2.15E+01 |  |
| H3♀ | 13 | S2 |  | 0 | Y5 | Shanghai zoo | 4 | 7 | -2.37E+01 |  |
| PW15♀ | 13 | PWB | Minshan Pingwu | 0 | PW14 | Minshan Pingwu | 1 | 1 | -1.15E+00 |  |
| PW15♀ | 13 | PWB |  | 0 | PW16 | Minshan Pingwu | 3 | 3 | -8.66E+00 |  |
| PW15♀ | 13 | PWB |  | 0 | PW19 | Minshan Pingwu | 2 | 3 | -7.92E+00 |  |
| PW15♀ | 13 | PWB |  | 0 | PW3 | Minshan Pingwu | 3 | 3 | -1.07E+01 |  |
| PW15♀ | 13 | PWB |  | 0 | PW7 | Minshan Pingwu | 3 | 3 | -9.34E+00 |  |
| PW15♀ | 13 | PWB |  | 0 | PW9 | Minshan Pingwu | 3 | 3 | -1.07E+01 |  |
| PW15♀ | 13 | PWB |  | 0 | PWA | Minshan Pingwu | 0 | 0 | 2.26E+00 | - |
| PW15♀ | 13 | PWB |  | 0 | PWF | Minshan Pingwu | 1 | 1 | 2.10E+00 |  |
| PW15♀ | 13 | PWB |  | 0 | PWG | Minshan Pingwu | 2 | 2 | -7.64E+00 |  |

Trio confidence: * for strict confidence, + for relaxed confidence, and - for a most likely candidate parent not assigned parentage.

R F= Recorded Father

Supplementary Table 4d. Paternity test with 12 loci.

| Offspring ID | Loci Typed | Candidate Mother ID | Population From | Pair Loci Mismatching | Candidate Father ID | Population From | Pair Loci Mismatching | Trio Loci Mismatching | Trio LOD Score | Trio Confidence |
| --- | --- | --- | --- | --- | --- | --- | --- | --- | --- | --- |
| G1 ♀ | 12 | G2 | Shennongjia | 0 | A180 | Minshan Shehong | 1 | 5 | -1.29E+01 |  |
| G1 ♀ | 12 | G2 |  | 0 | A230 | No record | 2 | 6 | -1.76E+01 |  |
| G1 ♀ | 12 | G2 |  | 0 | B27 | No record | 1 | 1 | 4.90E+00 |  |
| G1 ♀ | 12 | G2 |  | 0 | CC | Chengdu zoo | 0 | 0 | 1.01E+01 | **-** |
| G1 ♀ | 12 | G2 |  | 0 | C16 | Qionglai Longchi | 2 | 5 | -1.26E+01 |  |
| G1 ♀ | 12 | G2 |  | 0 | SW3 | Minshan Beichuan | 2 | 6 | -1.77E+01 |  |
| G1 ♀ | 12 | G2 |  | 0 | W2 | Qionglai Heishuihe | 6 | 8 | -2.65E+01 |  |
| G1 ♀ | 12 | G2 |  | 0 | Y3 | Minshan Xindu | 3 | 5 | -1.21E+01 |  |
| G1 ♀ | 12 | G2 |  | 0 | Y5 | Shanghai zoo | 3 | 6 | -1.86E+01 |  |
| G6 ♂ | 12 | J2 | Chengdu zoo | 0 | A180 | Minshan Shehong | 5 | 5 | -1.70E+01 |  |
| G6 ♂ | 12 | J2 |  | 0 | A230 | No record | 0 | 1 | 2.76E+00 |  |
| G6 ♂ | 12 | J2 |  | 0 | B27 | No record | 4 | 6 | -2.27E+01 |  |
| G6 ♂ | 12 | J2 |  | 0 | CC | Chengdu zoo | 0 | 3 | -7.80E+00 |  |
| G6 ♂ | 12 | J2 |  | 0 | C16 | Qionglai Longchi | 0 | 0 | 8.08E+00 | - |
| G6 ♂ | 12 | J2 |  | 0 | SW3 | Minshan Beichuan | 3 | 3 | -7.65E+00 |  |
| G6 ♂ | 12 | J2 |  | 0 | W2 | Qionglai Heishuihe | 3 | 4 | -1.01E+01 |  |
| G6 ♂ | 12 | J2 |  | 0 | Y3 | Minshan Xindu | 4 | 6 | -2.05E+01 |  |
| G6 ♂ | 12 | J2 |  | 0 | Y5 | Shanghai zoo | 4 | 7 | -2.45E+01 |  |
| J5 ♂ | 12 | J2 | Chengdu zoo | 0 | A180 | Minshan Shehong | 6 | 6 | -1.98E+01 |  |
| J5 ♂ | 12 | J2 |  | 0 | A230 | No record | 1 | 1 | 4.46E+00 |  |
| J5 ♂ | 12 | J2 |  | 0 | B27 | No record | 2 | 3 | -6.82E+00 |  |
| J5 ♂ | 12 | J2 |  | 0 | CC | Chengdu zoo | 0 | 2 | -3.72E-01 |  |
| J5 ♂ | 12 | J2 |  | 0 | C16 | Qionglai Longchi | 0 | 0 | 9.51E+00 | - |
| J5 ♂ | 12 | J2 |  | 0 | SW3 | Minshan Beichuan | 5 | 5 | -1.45E+01 |  |
| J5 ♂ | 12 | J2 |  | 0 | W2 | Qionglai Heishuihe | 4 | 4 | -8.71E+00 |  |
| J5 ♂ | 12 | J2 |  | 0 | Y3 | Minshan Xindu | 3 | 4 | -9.12E+00 |  |
| J5 ♂ | 12 | J2 |  | 0 | Y5 | Shanghai zoo | 3 | 4 | -9.63E+00 |  |
| Z1♀ | 12 | S2 | Chengdu zoo | 0 | A180 | Minshan Shehong | 0 | 0 | 4.89E+00 | - |
| Z1♀ | 12 | S2 |  | 0 | A230 | No record | 1 | 2 | -2.67E+00 |  |
| Z1♀ | 12 | S2 |  | 0 | B27 | No record | 1 | 2 | -3.18E+00 |  |
| Z1♀ | 12 | S2 |  | 0 | CC | Chengdu zoo | 1 | 2 | -4.02E+00 |  |
| Z1♀ | 12 | S2 |  | 0 | C16 | Qionglai Longchi | 4 | 4 | -1.17E+01 |  |
| Z1♀ | 12 | S2 |  | 0 | SW3 | Minshan Beichuan | 2 | 2 | -2.80E+00 |  |
| Z1♀ | 12 | S2 |  | 0 | W2 | Qionglai Heishuihe | 4 | 4 | -1.12E+01 |  |
| Z1♀ | 12 | S2 |  | 0 | Y3 | Minshan Xindu | 3 | 4 | -1.22E+01 |  |
| Z1♀ | 12 | S2 |  | 0 | Y5 | Shanghai zoo | 4 | 4 | -1.11E+01 |  |
| Y1♂ | 12 | S1 | Chengdu zoo | 0 | A180 | Minshan Shehong | 5 | 5 | -1.67E+01 |  |
| Y1♂ | 12 | S1 |  | 0 | A230 | No record | 2 | 3 | -7.55E+00 |  |
| Y1♂ | 12 | S1 |  | 0 | B27 | No record | 0 | 0 | 4.76E+00 | - (error) |
| Y1♂ | 12 | S1 |  | 0 | CC | Chengdu zoo | 0 | 0 | 4.04E+00 | (R F) |
| Y1♂ | 12 | S1 |  | 0 | C16 | Qionglai Longchi | 4 | 5 | -1.57E+01 |  |
| Y1♂ | 12 | S1 |  | 0 | SW3 | Minshan Beichuan | 4 | 4 | -1.31E+01 |  |
| Y1♂ | 12 | S1 |  | 0 | W2 | Qionglai Heishuihe | 5 | 6 | -1.97E+01 |  |
| Y1♂ | 12 | S1 |  | 0 | Y3 | Minshan Xindu | 1 | 1 | 2.59E+00 |  |
| Y1♂ | 12 | S1 |  | 0 | Y5 | Shanghai zoo | 2 | 5 | -1.53E+01 |  |
| H3♀ | 12 | S2 | Chengdu zoo | 0 | A180 | Minshan Shehong | 0 | 0 | 1.00E+01 | - |
| H3♀ | 12 | S2 |  | 0 | A230 | No record | 2 | 5 | -1.57E+01 |  |
| H3♀ | 12 | S2 |  | 0 | B27 | No record | 2 | 5 | -1.51E+01 |  |
| H3♀ | 12 | S2 |  | 0 | CC | Chengdu zoo | 3 | 6 | -1.98E+01 |  |
| H3♀ | 12 | S2 |  | 0 | C16 | Qionglai Longchi | 5 | 7 | -2.40E+01 |  |
| H3♀ | 12 | S2 |  | 0 | SW3 | Minshan Beichuan | 2 | 4 | -1.08E+01 |  |
| H3♀ | 12 | S2 |  | 0 | W2 | Qionglai Heishuihe | 5 | 6 | -1.82E+01 |  |
| H3♀ | 12 | S2 |  | 0 | Y3 | Minshan Xindu | 3 | 6 | -1.78E+01 |  |
| H3♀ | 12 | S2 |  | 0 | Y5 | Shanghai zoo | 4 | 6 | -2.00E+01 |  |
| PW15♀ | 12 | PWB | Minshan Pingwu | 0 | PW14 | Minshan Pingwu | 1 | 1 | -1.66E+00 |  |
| PW15♀ | 12 | PWB |  | 0 | PW16 | Minshan Pingwu | 3 | 3 | -9.17E+00 |  |
| PW15♀ | 12 | PWB |  | 0 | PW19 | Minshan Pingwu | 2 | 3 | -8.43E+00 |  |
| PW15♀ | 12 | PWB |  | 0 | PW3 | Minshan Pingwu | 3 | 3 | -1.12E+01 |  |
| PW15♀ | 12 | PWB |  | 0 | PW7 | Minshan Pingwu | 3 | 3 | -9.85E+00 |  |
| PW15♀ | 12 | PWB |  | 0 | PW9 | Minshan Pingwu | 3 | 3 | -1.05E+01 |  |
| PW15♀ | 12 | PWB |  | 0 | PWA | Minshan Pingwu | 0 | 0 | 1.75E+00 | (R F) |
| PW15♀ | 12 | PWB |  | 0 | PWF | Minshan Pingwu | 0 | 0 | 5.85E+00 | -(error) |
| PW15♀ | 12 | PWB |  | 0 | PWG | Minshan Pingwu | 2 | 2 | -7.47E+00 |  |

Trio confidence: * for strict confidence, + for relaxed confidence, and - for a most likely candidate parent not assigned parentage.

R F= Recorded Father

Supplementary Table 4e. Paternity test with 7 loci.

| Offspring ID | Loci Typed | Candidate Mother ID | Population From | Pair Loci Mismatching | Candidate Father ID | Population From | Pair Loci Mismatching | Trio Loci Mismatching | Trio LOD Score | Trio Confidence |
| --- | --- | --- | --- | --- | --- | --- | --- | --- | --- | --- |
| G1 ♀ | 7 | G2 | Shennongjia | 0 | A180 | Minshan Shehong | 1 | 5 | -1.32E+01 |  |
| G1 ♀ | 7 | G2 |  | 0 | A230 | No record | 2 | 5 | -1.39E+01 |  |
| G1 ♀ | 7 | G2 |  | 0 | B27 | No record | 2 | 2 | 6.37E-01 |  |
| G1 ♀ | 7 | G2 |  | 0 | CC | Chengdu zoo | 0 | 0 | 9.15E+00 | - |
| G1 ♀ | 7 | G2 |  | 0 | C16 | Qionglai Longchi | 3 | 5 | -1.43E+01 |  |
| G1 ♀ | 7 | G2 |  | 0 | SW3 | Minshan Beichuan | 3 | 7 | -2.26E+01 |  |
| G1 ♀ | 7 | G2 |  | 0 | W2 | Qionglai Heishuihe | 4 | 6 | -1.85E+01 |  |
| G1 ♀ | 7 | G2 |  | 0 | Y3 | Minshan Xindu | 2 | 3 | -4.21E+00 |  |
| G1 ♀ | 7 | G2 |  | 0 | Y5 | Shanghai zoo | 3 | 4 | -9.95E+00 |  |
| G6 ♂ | 7 | J2 | Chengdu zoo | 0 | A180 | Minshan Shehong | 4 | 4 | -1.31E+01 |  |
| G6 ♂ | 7 | J2 |  | 0 | A230 | No record | 1 | 2 | -3.37E+00 |  |
| G6 ♂ | 7 | J2 |  | 0 | B27 | No record | 4 | 7 | -2.57E+01 |  |
| G6 ♂ | 7 | J2 |  | 0 | CC | Chengdu zoo | 0 | 4 | -1.28E+01 |  |
| G6 ♂ | 7 | J2 |  | 0 | C16 | Qionglai Longchi | 0 | 0 | 7.83E+00 | - |
| G6 ♂ | 7 | J2 |  | 0 | SW3 | Minshan Beichuan | 4 | 4 | -1.24E+01 |  |
| G6 ♂ | 7 | J2 |  | 0 | W2 | Qionglai Heishuihe | 2 | 3 | -7.63E+00 |  |
| G6 ♂ | 7 | J2 |  | 0 | Y3 | Minshan Xindu | 3 | 6 | -2.09E+01 |  |
| G6 ♂ | 7 | J2 |  | 0 | Y5 | Shanghai zoo | 2 | 5 | -1.57E+01 |  |
| J5 ♂ | 7 | J2 | Chengdu zoo | 1 (GSM47) | A180 | Minshan Shehong | 5 | 5 | -1.82E+01 |  |
| J5 ♂ | 7 | J2 |  | 1 | A230 | No record | 2 | 2 | -4.51E+00 |  |
| J5 ♂ | 7 | J2 |  | 1 | B27 | No record | 3 | 4 | -1.31E+01 |  |
| J5 ♂ | 7 | J2 |  | 1 | CC | Chengdu zoo | 1 | 3 | -8.66E+00 |  |
| J5 ♂ | 7 | J2 |  | 1 | C16 | Qionglai Longchi | 0 | 1 | 2.49E+00 | - |
| J5 ♂ | 7 | J2 |  | 1 | SW3 | Minshan Beichuan | 6 | 6 | -2.22E+01 |  |
| J5 ♂ | 7 | J2 |  | 1 | W2 | Qionglai Heishuihe | 3 | 3 | -8.46E+00 |  |
| J5 ♂ | 7 | J2 |  | 1 | Y3 | Minshan Xindu | 3 | 4 | -1.29E+01 |  |
| J5 ♂ | 7 | J2 |  | 1 | Y5 | Shanghai zoo | 1 | 3 | -7.51E+00 |  |
| Z1♀ | 7 | S2 | Chengdu zoo | 0 | A180 | Minshan Shehong | 1 | 1 | -2.07E-01 | (R F) |
| Z1♀ | 7 | S2 |  | 0 | A230 | No record | 0 | 1 | 8.28E-01 | -(error) |
| Z1♀ | 7 | S2 |  | 0 | B27 | No record | 2 | 3 | -8.95E+00 |  |
| Z1♀ | 7 | S2 |  | 0 | CC | Chengdu zoo | 0 | 1 | -1.22E+00 |  |
| Z1♀ | 7 | S2 |  | 0 | C16 | Qionglai Longchi | 4 | 4 | -1.34E+01 |  |
| Z1♀ | 7 | S2 |  | 0 | SW3 | Minshan Beichuan | 2 | 2 | -3.79E+00 |  |
| Z1♀ | 7 | S2 |  | 0 | W2 | Qionglai Heishuihe | 2 | 2 | -3.60E+00 |  |
| Z1♀ | 7 | S2 |  | 0 | Y3 | Minshan Xindu | 1 | 2 | -3.81E+00 |  |
| Z1♀ | 7 | S2 |  | 0 | Y5 | Shanghai zoo | 1 | 1 | 7.96E-01 |  |
| Y1♂ | 7 | S1 | Chengdu zoo | 0 | A180 | Minshan Shehong | 4 | 4 | -1.34E+01 |  |
| Y1♂ | 7 | S1 |  | 0 | A230 | No record | 2 | 4 | -1.33E+01 |  |
| Y1♂ | 7 | S1 |  | 0 | B27 | No record | 0 | 0 | 5.90E+00 |  |
| Y1♂ | 7 | S1 |  | 0 | CC | Chengdu zoo | 0 | 0 | 3.17E+00 | (R F) |
| Y1♂ | 7 | S1 |  | 0 | C16 | Qionglai Longchi | 5 | 6 | -2.20E+01 |  |
| Y1♂ | 7 | S1 |  | 0 | SW3 | Minshan Beichuan | 5 | 5 | -1.80E+01 |  |
| Y1♂ | 7 | S1 |  | 0 | W2 | Qionglai Heishuihe | 3 | 5 | -1.71E+01 |  |
| Y1♂ | 7 | S1 |  | 0 | Y3 | Minshan Xindu | 0 | 0 | 6.59E+00 | -(error) |
| Y1♂ | 7 | S1 |  | 0 | Y5 | Shanghai zoo | 2 | 4 | -1.23E+01 |  |
| H3♀ | 7 | S2 | Chengdu zoo | 0 | A180 | Minshan Shehong | 0 | 0 | 8.44E+00 | - |
| H3♀ | 7 | S2 |  | 0 | A230 | No record | 1 | 4 | -1.26E+01 |  |
| H3♀ | 7 | S2 |  | 0 | B27 | No record | 2 | 3 | -7.29E+00 |  |
| H3♀ | 7 | S2 |  | 0 | CC | Chengdu zoo | 2 | 5 | -1.70E+01 |  |
| H3♀ | 7 | S2 |  | 0 | C16 | Qionglai Longchi | 5 | 6 | -2.16E+01 |  |
| H3♀ | 7 | S2 |  | 0 | SW3 | Minshan Beichuan | 1 | 2 | -2.12E+00 |  |
| H3♀ | 7 | S2 |  | 0 | W2 | Qionglai Heishuihe | 3 | 5 | -1.63E+01 |  |
| H3♀ | 7 | S2 |  | 0 | Y3 | Minshan Xindu | 2 | 4 | -1.11E+01 |  |
| H3♀ | 7 | S2 |  | 0 | Y5 | Shanghai zoo | 4 | 5 | -1.62E+01 |  |
| PW15♀ | 7 | PWB | Minshan Pingwu | 0 | PW14 | Minshan Pingwu | 1 | 1 | -2.05E+00 |  |
| PW15♀ | 7 | PWB |  | 0 | PW16 | Minshan Pingwu | 1 | 2 | -5.72E+00 |  |
| PW15♀ | 7 | PWB |  | 0 | PW19 | Minshan Pingwu | 0 | 2 | -5.57E+00 |  |
| PW15♀ | 7 | PWB |  | 0 | PW3 | Minshan Pingwu | 0 | 1 | -1.74E+00 |  |
| PW15♀ | 7 | PWB |  | 0 | PW7 | Minshan Pingwu | 1 | 2 | -5.73E+00 |  |
| PW15♀ | 7 | PWB |  | 0 | PW9 | Minshan Pingwu | 0 | 1 | -2.43E+00 |  |
| PW15♀ | 7 | PWB |  | 0 | PWA | Minshan Pingwu | 0 | 0 | 1.56E+00 | - |
| PW15♀ | 7 | PWB |  | 0 | PWF | Minshan Pingwu | 1 | 2 | -5.99E+00 |  |
| PW15♀ | 7 | PWB |  | 0 | PWG | Minshan Pingwu | 0 | 1 | -3.79E+00 |  |

Trio confidence: * for strict confidence, + for relaxed confidence, and - for a most likely candidate parent not assigned parentage.

R F= Recorded Father
