# Supplementary material for: Genome-wide screening of microsatellites in golden snub-nosed monkey (Rhinopithecus roxellana), for the development of a standardized genetic marker system
Source: Sci Rep. 2020 Jun 30;10:10614. doi: 10.1038/s41598-020-67451-2 (PMC7326997; doi:10.1038/s41598-020-67451-2)
Supplement: Supplementary file 5 — Supplementary file5 (DOCX 16 kb) [file 41598_2020_67451_MOESM5_ESM.docx]

Supplementary Table 5. Mismatched loci between recorded parents and offspring in 14 loci paternity test.

| Mismatched Loci | Offspring ID and sample type | Genotype | Mother ID and smaples type | Genotype | Father ID and samples type | ather Genotype |
| --- | --- | --- | --- | --- | --- | --- |
| GSM47 | J5 ♂ (fecal sample) | **163 163** | J2 (fecal sample) | **159 167** | C16 (blood sample) | 163 163 |
| GSM05 | J5 ♂ (fecal sample) | **145 145** | J2 (fecal sample) | 145 149 | C16 (blood sample) | **149 149** |
|  |  |  |  |  |  |  |
| GSM47 | Z1♀ (fecal sample) | **159 159** | S2 (muscle sample ) | 159 167 | A180 (fecal & blood sample) | **155 155** |
| GSM05 | Z1♀ (fecal sample) | **149 149** | S2 (muscle samples) | **145 145** | A180 (fecal & blood sample) | **145 145** |
