# Supplementary material for: Genome-wide screening of microsatellites in golden snub-nosed monkey (Rhinopithecus roxellana), for the development of a standardized genetic marker system
Source: Sci Rep. 2020 Jun 30;10:10614. doi: 10.1038/s41598-020-67451-2 (PMC7326997; doi:10.1038/s41598-020-67451-2)
Supplement: Supplementary file 6 — Supplementary file6 (DOC 29 kb) [file 41598_2020_67451_MOESM6_ESM.doc]

**Genome-wide screening of microsatellites in golden snub-nosed monkey (*Rhinopithecus roxellana*), for the development of a standardized genetic marker system.**

YanSen Cai^1,2^, HaoYang Yu^1^, Hua Liu^1^, Cong Jiang^1^, Ling Sun^4^, LiLi Niu^3^, XuanZhen Liu^3^, DaYong Li^4^, Jing Li^1*^

1. Key Laboratory of Bio-resources and Eco-environment (Ministry of Education), Sichuan Key Laboratory of Conservation Biology on Endangered Wildlife, College of Life Sciences, Sichuan University, Chengdu 610064, P.R. China.

2. Department of Cell Biology and Genetic, School of Basic Medical Sciences, Southwest Medical University, Luzhou 646000, P.R. China.

3. Chengdu Zoo, Chengdu 610064, P.R. China.

4. College of Life Sciences, China West Normal University, Nanchong, 637000, P.R. China.

Corresponding author: Jing Li, Key Laboratory of Bio-resources and Eco-environment (Ministry of Education), College of Life Sciences, Sichuan University, Chengdu 610064, P.R. China. Fax: +86 28 85414886, e-mail: [ljtjf@126.com](mailto:ljtjf@126.com), telephone number: 13808067169.

Supplementary Table 1. The 12 resequencing genomes used to develop the standardized STR system.

Supplementary Table 2. The tests on the relationship between the exposure time of fecal samples and the stability of the 16 loci.

Supplementary Table 3. A standardized marker system data base for golden snub-nosed monkeys in Chengdu Zoo and Pingwu.

Supplementary Table 4a, 4b, 4c, 4d and 4e paternity tests with different loci combinations.

Supplementary Table 5. Mismatched loci between recorded parents and offspring.
